# Heterogeneous Expression and Subcellular Localization of Pyruvate Dehydrogenase Complex in Prostate Cancer

**DOI:** 10.3389/fonc.2022.873516

**Published:** 2022-05-25

**Authors:** Caroline E. Nunes-Xavier, Janire Mingo, Maite Emaldi, Karine Flem-Karlsen, Gunhild M. Mælandsmo, Øystein Fodstad, Roberto Llarena, José I. López, Rafael Pulido

**Affiliations:** ^1^ Biomarkers in Cancer, Biocruces Bizkaia Health Research Institute, Barakaldo, Spain; ^2^ Department of Tumor Biology, Institute for Cancer Research, Oslo University Hospital Radiumhospitalet, Oslo, Norway; ^3^ Department of Urology, Cruces University Hospital, Barakaldo, Spain; ^4^ Department of Pathology, Cruces University Hospital, Barakaldo, Spain; ^5^ Ikerbasque, Basque Foundation for Science, Bilbao, Spain

**Keywords:** prostate cancer (PCa), pyruvate dehydrogenase (PDH), pyruvate dehydrogenase kinase (PDK), androgen receptor (AR), dichloroacetate (DCA)

## Abstract

**Background:**

Pyruvate dehydrogenase (PDH) complex converts pyruvate into acetyl-CoA by pyruvate decarboxylation, which drives energy metabolism during cell growth, including prostate cancer (PCa) cell growth. The major catalytic subunit of PDH, PDHA1, is regulated by phosphorylation/dephosphorylation by pyruvate dehydrogenase kinases (PDKs) and pyruvate dehydrogenase phosphatases (PDPs). There are four kinases, PDK1, PDK2, PDK3 and PDK4, which can phosphorylate and inactivate PDH; and two phosphatases, PDP1 and PDP2, that dephosphorylate and activate PDH.

**Methods:**

We have analyzed by immunohistochemistry the expression and clinicopathological correlations of PDHA1, PDP1, PDP2, PDK1, PDK2, PDK3, and PDK4, as well as of androgen receptor (AR), in a retrospective PCa cohort of patients. A total of 120 PCa samples of representative tumor areas from all patients were included in tissue microarray (TMA) blocks for analysis. In addition, we studied the subcellular localization of PDK2 and PDK3, and the effects of the PDK inhibitor dichloroacetate (DCA) in the growth, proliferation, and mitochondrial respiration of PCa cells.

**Results:**

We found heterogeneous expression of the PDH complex components in PCa tumors. PDHA1, PDP1, PDK1, PDK2, and PDK4 expression correlated positively with AR expression. A significant correlation of PDK2 immunostaining with biochemical recurrence and disease-free survival was revealed. In PCa tissue specimens, PDK2 displayed cytoplasmic and nuclear immunostaining, whereas PDK1, PDK3 and PDK4 showed mostly cytoplasmic staining. In cells, ectopically expressed PDK2 and PDK3 were mainly localized in mitochondria compartments. An increase in maximal mitochondrial respiration was observed in PCa cells upon PDK inhibition by DCA, in parallel with less proliferative capacity.

**Conclusion:**

Our findings support the notion that expression of specific PDH complex components is related with AR signaling in PCa tumors. Furthermore, PDK2 expression associated with poor PCa prognosis. This highlights a potential for PDH complex components as targets for intervention in PCa.

## Introduction

PCa is a long-latency cancer, evolving from low malignancy early stages (prostatic intraepithelial neoplasia) to high-grade and metastatic adenocarcinomas, which frequently do not respond to anti-androgen hormone therapies (castrate-resistant prostate cancer, CRPC) (1–3). The androgen pathway is the central signaling pathway in PCa, together with the retinoblastoma (RB), PI3K/PTEN/AKT/mTOR, and RAS/RAF/MAPK pathways (4–7). Frequent alterations in PCa include gene amplification of MYC transcription factor and androgen receptor (AR), the gene deletion of NKX3.1 homeobox, RB1, and PTEN phosphatase, and the gene reorganization of the ETS family of transcription factors (8–12). Currently, the identification of early tumor markers, including metabolic biomarkers, and molecular targets for effective PCa treatment is a research priority (13–17).

PCa presents a high extent of metabolic modifications, mainly related with increase in aerobic glycolysis and protein and fatty acid synthesis (18, 19). As in other cancer types, this metabolic switch facilitates the synthesis of biomolecules required by the tumor cell to support its rapid growth and division (20). PCa cells display high levels of aerobic glycolysis in the more advanced tumor stages, while primary PCa cells show higher oxidative respiration than non-transformed prostatic cells. This is mainly due to a decrease in Zn accumulation in primary PCa cells, which allows citrate oxidation through the Krebs cycle. High *de novo* fatty acid synthesis is characteristic of PCa progression towards CRPC, which is facilitated by high expression of fatty acid synthase (FASN) and other lipogenic enzymes (21, 22). PCa progression and metastasis has been recently linked to glycolytic enzymes such as pyruvate kinase isoform M2 (PKM2) (23). Together, these observations suggest that specific interference with key metabolic reactions could be useful to improve the current therapies for advanced PCa.

The enzyme pyruvate dehydrogenase (PDH) is essential in the glycolytic and Krebs cycle metabolism, and play important roles in carcinogenesis, making this enzyme a feasible therapeutic target in cancer (24–27). PDH exists as a multi-enzyme complex formed by three catalytic (E1 [two genes: *PDHA1*-*2*], E2 [*DLAT*], and E3 [*DLD*]) and three regulatory subunits (E3BP [*PDHX*], PDKs [four genes: *PDK1-4*], and PDPs [two genes: *PDP1-2*). The mRNA expression patterns of these genes in prostate tissues and prostate tumors are distinct, as shown in databases GTEx (Genotype-Tissue Expression; https://gtexportal.org) and TCGA (The Cancer Genome Atlas; https://www.proteinatlas.org), but comprehensive comparative studies on the expression at the protein level of these enzymes in PCa are lacking. The association of PDKs expression with poor prognosis and resistance to anti-cancer therapies is widely documented, and PDKs inhibition (which results in PDH activation) constitutes a potential therapeutic possibility in several cancer types, including PCa (28–34). In addition, differing results have been reported on the association of other PDH components, such as PDHA1 and PDP1, with PCa prognosis (35, 36). Together, this makes relevant to investigate comparatively the individual expression and function of the distinct components of the PDH complex in relation with PCa progression and malignancy.

In this study, we have evaluated the expression and subcellular localization of components of the PDH complex in PCa, including PDHA1, PDP1, PDP2, PDK1, PDK2, PDK3 and PDK4. We have found specific correlations between the expression of some of these PDH complex components and AR expression in PCa tumors. Furthermore, a significant correlation of PDK2 PCa tumor immunostaining with patient biochemical recurrence and disease-free survival has been revealed. We discuss the potential of PDH complex components as targets for intervention in PCa.

## Material and Methods

### Cell Lines

Simian kidney COS-7 cells were cultured in DMEM (Dulbecco’s Modified Eagle’s Medium) (Lonza, Basel, Switzerland) medium supplemented with 5% FBS (Fetal Bovine Serum) (Sigma Aldrich, St. Louis, MO, USA). Human prostate carcinoma LNCaP cells were cultured in RPMI-1640 (Lonza) medium supplemented with 10% FBS. Human prostate carcinoma DU-145 cells were cultured in EMEM (Eagle’s Minimal Essential Medium) (Lonza) medium supplemented with 10% FBS. All media were supplemented with 1% L-Glutamine and 1% penicillin/streptomycin (Lonza). Cells were incubated at 37°C and 5% CO2.

### Plasmids, Transfection, and Immunoblot

Human PDK2 (NM_002611.4) and PDK3 (NM_001142386) cDNAs, cloned in pcDNA3.1+/C-DYK mammalian expression plasmids (C-terminal Flag fusion), were purchased from GeneScript (Piscataway, NJ, USA). pRK5 Flag-PTEN was made by PCR incorporation of an N-terminal Flag sequence to human PTEN (NM_000314) from pRK5 PTEN (37). Cells were transiently transfected with empty vector, pRK5 Flag-PTEN, pCDNA3.1 PDK2-Flag, or pCDNA3.1 PDK3-Flag using GenJet reagent (SignaGen, Frederick, MD, USA). Cells were lysed in M-PER extraction reagent (ThermoFisher, Waltham, MA, USA) and processed for immunoblot as described (38). Primary antibody used was mouse anti-Flag (1:500, MAB3118, Sigma Aldrich). Secondary antibody was IRDye 680RD Goat anti-Mouse (LI-COR, Lincoln, NE, USA). Blots were processed with Odyssey CLx Imaging system (LI-COR).

### Metabolism/Seahorse

Oxygen Consumption Rate Assay Kit (Cayman Chemical, Ann Arbor, MI, USA) was used to measure extracellular oxygen consumption levels according to manufacturer’s instructions. XF96 Mitochondrial stress test was performed using Seahorse Extracellular Flux Analyzer XF96e (Agilent Technologies, Santa Clara, CA, USA) to measure the oxygen consumption rate (OCR) of cells according to manufacturer’s instructions. Seahorse assays were performed in at least triplicate wells in three independent experiments for each condition.

### Cell Proliferation and Confluence

Cell proliferation/viability of LNCaP and DU-145 cells was assessed as described (39). 5x10^3^ cells/well were plated in 96-well culture plates. A day after plating the cells, different concentrations of dichloroacetate (DCA; Sigma Aldrich) or vehicle were added. Cell proliferation was measured with the CellTiter 96^®^AQ_ueous_ One Solution Cell Proliferation Assay Kit (MTS Assay, Promega, Madison, WI, USA) in 96-well plates, and luminescence was measured at 490 nm using Victor3 microplate reader (PerkinElmer, Waltham MA, USA). To assess cell confluence, 5x10^3^ cells/well were seeded on 96-well plates and the cell confluence was measured every three hours by the IncuCyte FLR imaging microscopes (Essen Biosciences, Ann Arbor, MI, USA), as described (40). The cells were treated with the indicated DCA concentrations 21 h post-plating and were scanned for 72 h after adding the drug.

### Immunofluorescence

3x10^4^ COS-7 cells per well were plated in 8-well chamber slides for immunofluorescence (Ibidi, Gräfelfing, Germany). Transient transfection was performed as described above. Cells were washed and mitochondria were stained with Mitotracker™ Red CMXRos following manufacturer’s instructions (250 nM, 20 min) (ThermoFisher). before they were fixed in methanol for 5 min at -20°C and blocked in blocking solution (Phosphate Buffered Saline (PBS) containing 3% Bovine Serum Albumin (BSA). Mouse anti-Flag primary antibody (1/100 in blocking solution) was incubated overnight at 4°C in a wet chamber. Subsequently, cells were washed three times with PBS-BSA for 10 min prior to incubation with anti-mouse FITC secondary antibody (1/100) for 1 h in a wet chamber and darkness at room temperature. Cells were washed and mounted in Mounting Medium with DAPI (4’6-diamidino-2-phenylindole) (Abcam) and visualized by standard [NIKON ECLIPSE TE2000 (Nikon, Tokyo, Japan)] or confocal microscopy [ZEISS LSM880 AIRYSCAN (Zeiss, Jena, Germany)].

### Clinical Data and Tumor Samples

The PCa cohort has been previously described (41). Briefly, it consisted of 120 PCa patients treated with radical prostatectomy at Cruces University Hospital (Barakaldo, Spain) between 2000 and 2005. An experienced pathologist (JIL) selected tumor areas with well‐preserved tissue, representative of the whole tumor, from formalin‐fixed and paraffin‐embedded (FFPE) tumor tissue blocks, and TMA blocks were made from these areas. 4 μm sections were made from the TMA blocks, one of which was stained with hematoxylin and eosin (H&E) to verify the presence of tumor content. Biochemical recurrence (BR) was defined as a Prostate-Specific Antigen (PSA) measurement equal to or greater than 0.4 ng/ml after surgery. Follow‐up has been recorded until October 1, 2016. Cancer of the Prostate Risk Assessment Postsurgical (CAPRA‐S) score was calculated according to its definition (42), that is, by combining preoperative PSA, Gleason grade, surgical margins, extracapsular extension, seminal vesicle invasion, and lymph node invasion.

### Immunohistochemistry and Scoring

Immunohistochemistry (IHC) was carried out using the following primary antibodies: PDHA1 (Sigma Aldrich, HPA047864, dilution: 1:10), PDP1 (Sigma Aldrich, HPA019081, dilution 1:10), PDP2 (Sigma Aldrich, HPA019950, dilution 1:65), PDK1 (Cell Signaling, Danvers, MA, USA, HPA027376, dilution: 1:120), PDK2 (Sigma Aldrich, HPA008287, dilution 1:25), PDK3 (Sigma Aldrich, HPA046583, dilution 1:50), PDK4 (Sigma Aldrich, HPA056731, dilution 1:100), and AR (SP107 ready to use, Ventana, Roche, Basel, Switzerland) antibodies. Antigen retrieval was performed at pH 6 and pH 9 using PT link system (Agilent Technologies). IHC immunostainings were performed in automated immunostainers (EnVision FLEX, Dako Autostainer Plus; Dako, Glostrup, Denmark and BenchMark Ultra, Ventana Medical Systems, Tucson, AZ, USA). Antibodies were incubated for 30 min, followed by secondary antibody incubation for 15 min using Goat Anti Mouse and Anti-rabbit Ig/HRP secondary antibodies (Dako), FLEX/HPR for 20 min, FLEX DAB/Sub Chromo for 10 min, and finally counterstaining with hematoxylin. Immunostainings were evaluated in tumor cells as negative (weak/no staining) or positive (medium/high staining). The analysis was performed using a Nikon Eclipse 80i microscope (Nikon, Tokyo, Japan).

### Statistical Analysis

Error bars in results represent ± standard deviation (S.D.). Cell data was analyzed by GraphPad Prism t Test Calculator (San Diego, CA, USA), where significance was calculated using two-tailed student t-test. p values smaller than 0.05 were considered significant and are indicated with an asterisk (*). All experiments were performed at least twice, and results shown are from one representative experiment. The SPSS version 23 software (SPSS Inc., Chicago, IL, USA) was used for statistical calculations of the clinical material. For all the experiments, any p value below 0.05 was considered statistically significant.

## Results

**Figure 1:**
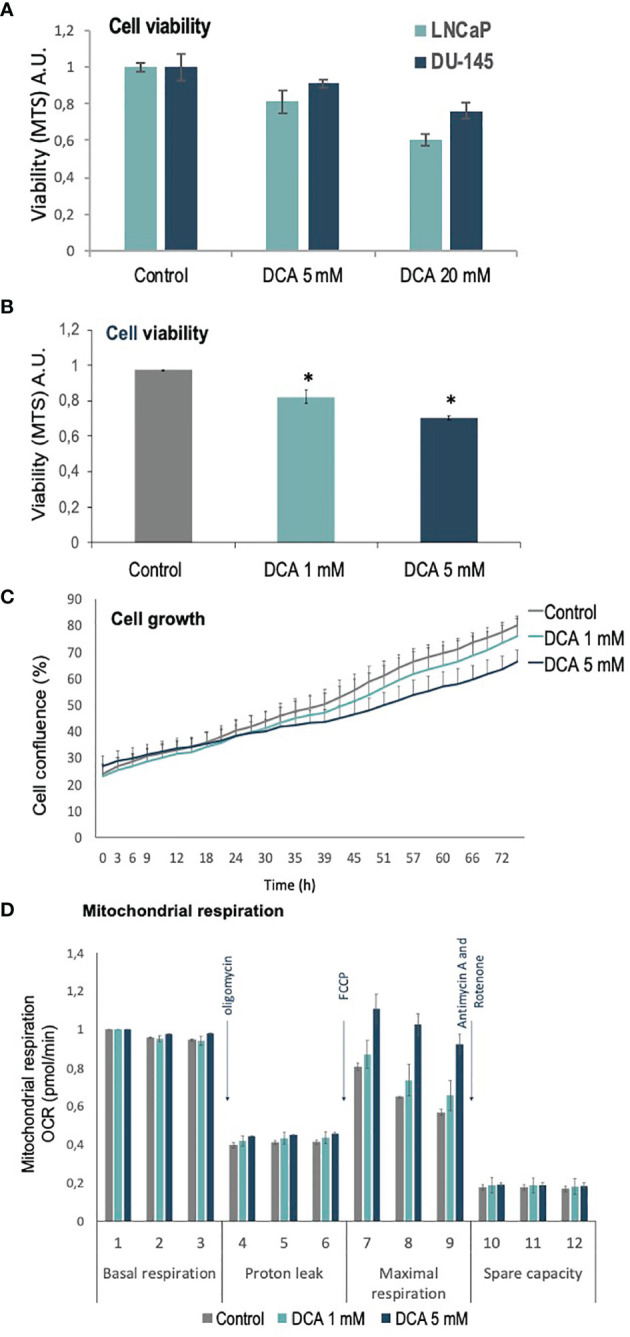
Viability, proliferation and mitochondrial function of PCa cells treated with DCA. **(A)** Cell viability is shown for LNCaP and DU-145 PCa cells, as determined by MTS analysis, after 72 h in the presence of DCA (5 mM and 20 mM). **(B)** Cell viability is shown for LNCaP cells, as determined by MTS analysis, after 72 h in the presence of DCA (1 mM and 5 mM). **(C)** Cell growth is shown for LNCaP cells, as determined by Incucyte live-cell analysis, after 72 h in the presence of DCA (1 mM and 5 mM). **(D)** Mitochondrial respiration is shown for LNCaP cells, as determined by Seahorse extracellular flux analysis, after 48 h in the presence of DCA (1 mM and 5 mM). p value below 0.05 are indicated with *.

PDH complex components, including the negative regulators of PDH activity, PDKs, have been involved in PCa carcinogenesis (27). We analyzed the role of PDKs in the growth, proliferation, and mitochondrial respiration of PCa cells, using the PDK inhibitor dichloroacetate (DCA), which selectively shifts the cancer cell metabolism from glycolysis to oxidative phosphorylation (29). As expected, DCA treatment inhibited in a dose-response manner the growth/viability of LNCaP and DU-145 PCa cells, as shown by MTS assay and cell confluence measurements (**Figures 1A–C**). In addition, an increase in maximal oxygen consumption rate (OCR) was observed in LNCaP PCa cells upon PDK inhibition by DCA, in parallel with less proliferative capacity and cell viability (**Figure 1D**). These results suggest a role for PDKs in the regulation of cell growth and viability of PCa cells.

**Figure 2:**
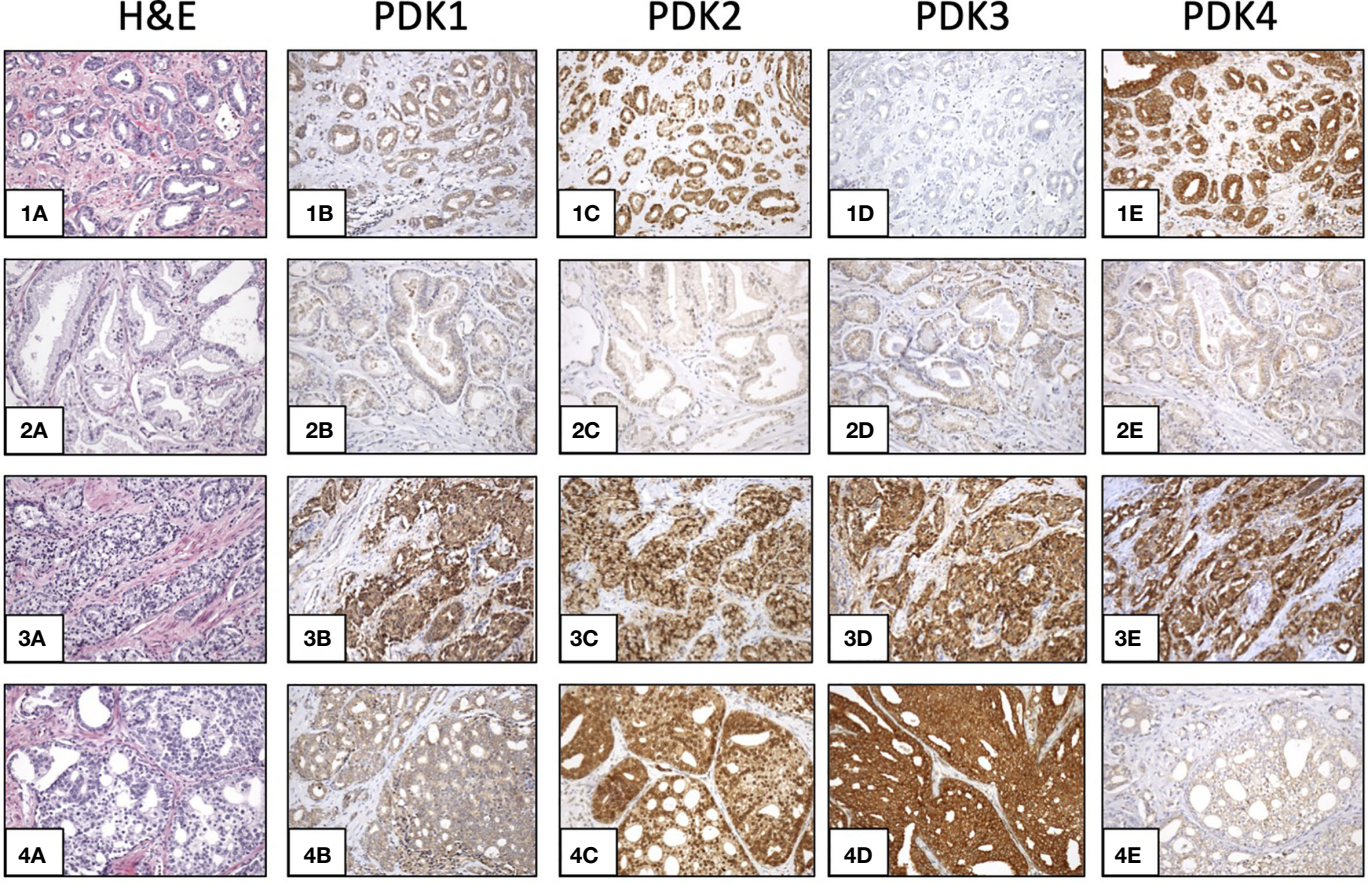
Expression of PDKs in PCa specimens. Immunohistochemical staining of expression of PDKs in four representative prostate carcinoma patient samples (1-4). Hematoxylin and eosin (H&E) staining (1A, 2A, 3A, 4A). High expression of all PDKs (case 3: 3B, 3C, 3D, 3E). Low expression of all PDKs (case 2: 2B, 2C, 2D, 2E). High expression of PDK2 and PDK4 (case 1: 1C, 1E), and low expression of PDK1 and PDK3 (case 1: 1B, 1D). High expression of PDK2 and PDK3 (case 4: 4C, 4D), and low expression of PDK1 and PDK4 (case 4: 4B, 4E). Magnification: X100.

**Figure 3:**
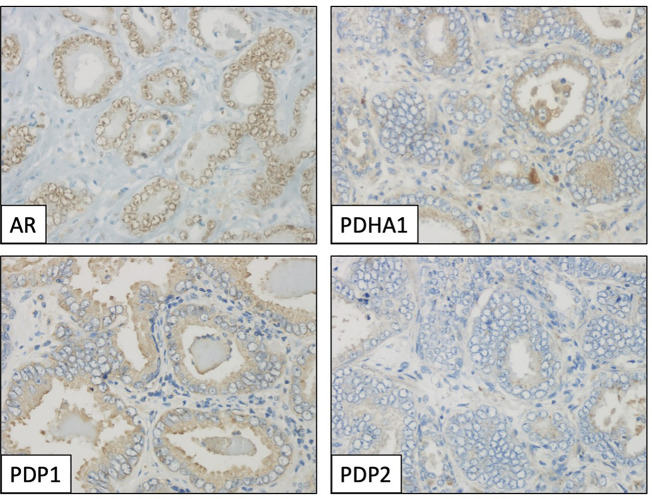
Immunohistochemical profile of a prostate adenocarcinoma specimen, showing positive staining for androgen receptor (AR, nuclear), PDHA1 and PDP1 (cytoplasmic), and negative for PDP2. Magnification: X400.

This prompted us to investigate the expression of PDKs and other PHD complex components in PCa patient tumor samples. The expression of PDHA1, PDP1, PDP2, PDK1, PDK2, PDK3, and PDK4, as well as the expression of AR was evaluated by IHC in a retrospective cohort of 120 PCa patients (**Tables 1**–**3**). FFPE samples from representative tumor areas were included in TMAs for analysis, and expression was scored as negative or positive. We observed heterogeneous expression of PDHA1, PDP1, PDP2, PDK1, PDK2, PDK3, and PDK4 in PCa specimens, and examples of different patterns of staining for the different PDKs are shown in **Figure 2**. PDK2 expression in tumors displayed a nuclear/cytoplasmic pattern, whereas PDK1, PDK3, and PDK4 expression was mostly cytoplasmic (**Figure 2**). PDP1 expression positively correlated with stage (p = 0.037) and extracapsular extension (p = 0.027) (**Table 1**). Importantly, we found a significant positive correlation of PDK2 immunostaining with biochemical recurrence (p = 0.033), and negative correlation with disease-free survival (p = 0.045), suggesting a negative prognostic role for PDK2 expression in PCa (**Table 2**). Significant positive correlations were found with respect to AR expression for PDHA1 (p = 0.035), PDP1 (p = 0.046), PDK1 (p = 0.003), PDK2 (p = 0.001), and PDK4 (p = 0.031) expression (**Table 3** and **Figure 3**). PDP2 and PDK3 did not show any significant correlation. Together, these findings show a heterogeneous expression pattern of PDH complex components in PCa related with AR and suggest an association between PDK2 expression and PCa progression.

**Table 1:** Correlation between clinical and pathological variables and PDHA1, PDP1 and PDP2 protein expression in prostate cancer.

Patients – no.	N = 120	PDHA1 negative		PDHA1 positive	PDP1 negative	PDP1 positive	PDP2 negative	PDP2 positive
		(N = 91)		(N = 28)	(N = 106)	(N = 13)	(N = 88)	(N = 31)
Median follow-up time (IQR) – year	120	ρ = -0.182 / P = 0.720			ρ = -0.124 / P = 0.745		ρ = -0.086 / P = 0.564	
	10.5 (9.8-12.4)	10.9 (1-16)		9.9 (5.9-15)	10.6 (1-16)	9.9 (5.9-15)	10.7 (1-16)	10 (5.9-15)
Median age at surgery (IQR) – year		ρ = 0.057 / P = 0.739			ρ = 0.041 / P = 0.615		ρ = 0.011 / P = 0.970	
	63 (59-68)	63 (48-71)		63.5 (52-73)	63 (48-73)	64 (53-69)	63 (48-73)	63 (50-70)
Age at surgery – no. (%)		ρ = 0.015 / P = 0.872			ρ = -0.027 / P = 0.767		ρ = 0.013 / P = 0.888	
< 65 year	78 (65)	60 (66)		18 (64)	69 (65)	9 (69)	58 (66)	20 (64.5)
> 65 year	42 (35)	31 (34)		10 (36)	37 (35)	4 (31)	30 (34)	11 (35.5)
Preoperative PSA – no. (%)		ρ = -0.100 / P = 0.440			ρ = -0.082 / P = 0.819		ρ = -0.168 / P = 0.311	
≤ 6 ng/ml	36 (30)	25 (27.5)		11 (39.5)	31 (29)	5 (38.5)	23 (26)	13 (42)
> 6 ng/ml and ≤ 10 ng/ml	43 (35)	34 (37.5)		8 (28.5)	37 (35)	5 (38.5)	32 (36.5)	11 (35.5)
> 10 ng/ml and ≤ 20 ng/ml	33 (27.5)	25 (27.5)		8 (28.5)	30 (28)	3 (23)	26 (29.5)	7 (22.5)
> 20 ng/ml	4 (3.3)	4 (4.5)		0 (0)	4 (4)	0 (0)	4 (4.5)	0 (0)
Missing	4 (3.3)	3 (3)		1 (3.5)	4 (4)	0 (0)	3 (3.5)	0 (0)
Gleason grade – no. (%)		ρ = -0.051 / P = 0.389			ρ = -0.004 / P = 0.545		ρ = 0.065 / P = 0.480	
≤ 6	72 (60)	55 (60.5)		16 (57)	64 (60)	7 (54)	55 (62.5)	16 (52)
3+4	22 (18)	14 (15.5)		8 (28.5)	18 (17)	4 (31)	14 (16)	8 (26)
4+3	7 (6)	6 (6.5)		1 (3.5)	7 (7)	0 (0)	6 (7)	1 (3)
≥ 8	19 (16)	16 (17.5)		3 (11)	17 (16)	2 (15)	13 (14.5)	6 (19)
Stage - no. (%)		ρ = 0.107 / P = 0.243			ρ = 0.191 / P = 0.037		ρ = - 0.024 / P = 0.797	
T2	99 (82.5)	77 (84.5)		21 (75)	90 (85)	8 (61.5)	72 (82)	26 (84)
T3	21 (17.5)	14 (15.5)		7 (25)	16 (15)	5 (38.5)	16 (18)	5 (16)
Surgical margins – no. (%)		ρ = -0.069 / P = 0.454			ρ = -0.084 / P = 0.360		ρ = -0.1 08 / P = 0.239	
Negative	78 (65)	58 (64)		20 (71.5)	68 (64)	10 (77)	55 (62.5)	23 (74)
Positive	42 (35)	33 (36)		8 (28.5)	38 (36)	3 (23)	33 (37.5)	8 (26)
Extracapsular extension– no. (%)		ρ = 0.122 / P = 0.185			ρ = 0.203 / P = 0.027		ρ = -0.011 / P = 0.907	
No	100 (83)	78 (86)		21 (75)	91 (86)	8 (61.5)	73 (83)	26 (84)
Yes	20 (17)	13 (14)		7 (25)	15 (14)	5 (38.5)	15 (17)	5 (16)
Seminal vesicle invasion – no. (%)		ρ = -0.017 / P = 0.849			ρ = 0.061 / P = 0.506		ρ = -0.124 / P = 0.175	
No	20 (17)	87 (96)		27 (96.5)	102 (96)	12 (92)	83 (94.5)	31 (100)
Yes	100 (83)	4 (4)		1 (3.5)	4 (4)	1 (8)	5 (5.5)	0 (0)
CAPRA-S risk group – no. (%)*		ρ = -0.111 / P = 0.529			ρ = -0.005 / P = 0.388		ρ = -0.1 45 / P = 0.240	
Low	48 (40)	35 (38.5)		13 (46.5)	44 (41.5)	4 (31)	36 (41)	12 (39)
Intermediate	44 (37)	34 (37)		9 (32)	37 (35)	6 (46)	34 (39)	9 (29)
High	9 (8)	8 (9)		1 (3.5)	9 (8.5)	0 (0)	9 (10)	0 (0)
Missing	19 (15)	14 (25.5)		5 (18)	16 (15)	3 (23)	9 (10)	10 (32)
Biochemical recurrence – no. (%)		ρ = -0.027 / P = 0.769			ρ = -0.084 / P = 0.360		ρ = 0.013 / P = 0.888	
Negative	78 (65)	59 (65)		19 (68)	68 (64)	10 (77)	58 (66)	20 (64.5)
Positve	42 (35)	32 (35)		9 (32)	38 (36)	3 (23)	30 (34)	11 (35.5)
Disease-free survival – no. (%)		ρ = 0.105 / P = 0.299			ρ = 0.074 / P = 0.463		ρ = -0.046 / P = 0.652	
Yes	41 (34)	32 (35)		8 (28)	37 (35)	3 (23)	28 (32)	12 (39)
No	58 (48)	41 (45)		17 (61)	51 (48)	7 (54)	43 (49)	15 (48)
Missing	21 (18)	18 (20)		5 (11)	18 (17)	3 (23)	17 (19)	4 (13)

*The CAPRA-S scores were categorized to give the three risk groups: Low risk if sscore 0-2; Intermediate risk if score 3 to 5; High risk if score 6 to 12.

Spearsman´s correlation ρ (95% CI) / P value.

IQR, interquartile range; PSA, prostate-specific antigen; AR, androgen receptor.

**Table 2:** Correlation between clinical and pathological variables and PDK1, PDK2, PDK3 and PDK4 protein expression in prostate cancer.

Patients – no.	N = 120	PDK1 negative	PDK1 positive	PDK2 negative	PDK2 positive	PDK3 negative	PDK3 positive	PDK4 negative	PDK4 positive
		(N = 26)	(N = 89)	(N = 17)	(N = 102)	(N = 13)	(N = 100)	(N = 14)	(N = 96)
Median follow-up time (IQR) – year	120	ρ = -0.180 / P = 0.295		ρ = 0.080 / P = 0.580		ρ = 0.006 / P = 0.592		ρ = -0.315 / P = 0.016	
	10.5 (9.8-12.4)	12 (8.4-14.9)	10.3 (1-16)	10 (2-14)	10.5 (1-16)	10.2 (9.4-13.9)	10.5 (1-16)	13.2 (1-15)	10.3 (2.1-16)
Median age at surgery (IQR) – year		ρ = 0.097 / P = 0.632		ρ = -0.112 / P = 0.603		ρ = 0.035 / P = 0.065		ρ = 0.019 / P = 0.834	
	63 (59-68)	62 (50-73)	63 (48-71)	64 (54-73)	63 (48-71)	61 (52-73)	63 (48-71)	62 (52-71)	63 (48-73)
Age at surgery – no. (%)		ρ = 0.046 / P = 0.625		ρ = -0.108 / P = 0.237		ρ = 0.022 / P = 0.817		ρ = 0.041 / P = 0.668	
< 65 year	78 (65)	18 (69)	57 (64)	9 (53)	69 (68)	9 (69)	66 (66)	10 (71)	63 (66)
> 65 year	42 (35)	8 (31)	32 (36)	8 (47)	33 (32)	4 (31)	34 (34)	4 (29)	33 (34)
Preoperative PSA – no. (%)		ρ = 0047 / P = 0.305		ρ = 0.020 / P = 0.875		ρ = -0.056 / P = 0.715		ρ = 0.109 / P = 0.280	
≤ 6 ng/ml	36 (30)	10 (38.5)	26 (29)	5 (29.5)	31 (30.5)	4 (31)	32 (32)	7 (50)	29 (30)
> 6 ng/ml and ≤ 10 ng/ml	43 (35)	6 (23)	34 (38)	6 (35)	36 (35)	3 (23)	36 (36)	2 (14)	34 (36)
> 10 ng/ml and ≤ 20 ng/ml	33 (27.5)	9 (34.5)	23(26)	5 (29.5)	28 (27.5)	4 (31)	27 (27)	4 (29)	27 (28)
> 20 ng/ml	4 (3.3)	0 (0)	4 (5)	0 (0)	4 (4)	1 (7.5)	3 (3)	0 (0)	4 (4)
Missing	4 (3.3)	1 (4)	2 (2)	1 (6)	3 (3)	1 (7.5)	2 (2)	1 (7)	2 (2)
Gleason grade – no. (%)		ρ = 0.179 / P = 0.056		ρ = 0.092 / P = 0.617		ρ = 0.042 / P = 0.839		ρ = 0.075 / P = 0.199	
≤ 6	72 (60)	19 (73)	51 (57)	12 (70)	59 (58)	8 (62)	61 (61)	8 (57)	60 (62.5)
3+4	22 (18)	4 (15.5)	17 (19)	3 (18)	19 (18)	3 (23)	18 (18)	5 (36)	16 (16.5)
4+3	7 (6)	3 (11.5)	4 (5)	0 (0)	7 (7)	1 (7.5)	5 (5)	1 (7)	5 (5)
≥ 8	19 (16)	0 (0)	17 (19)	2 (12)	17 (17)	1 (7.5)	16 (16)	0 (0)	15 (16)
Stage - no. (%)		ρ = 0.083 / P = 0.371		ρ = 0.000 / P = 1		ρ = 0.095 / P = 0.315		ρ = 0.1*07* / P = 0.243	
T2	99 (82.5)	23 (88.5)	72 (81)	14 (82)	84 (82)	12 (92)	81 (81)	14 (100)	77 (80)
T3	21 (17.5)	3 (11.5)	17 (19)	3 (18)	18 (18)	1 (8)	19 (19)	0 (0)	19 (20)
Surgical margins – no. (%)		ρ = -0.042 / P = 0.654		ρ = -0.007 / P = 0.937		ρ = 0.022 / P = 0.817		ρ = -0.132 / P = 0.165	
Negative	78 (65)	16 (61.5)	59 (66)	11 (65)	67 (66)	9 (69)	66 (66)	7 (50)	66 (69)
Positive	42 (35)	10 (38.5)	30 (34)	6 (35)	35 (34)	4 (31)	34 (34)	7 (50)	30 (31)
Extracapsular extension– no. (%)		ρ = 0.073 / P = 0.437		ρ = -0.009 / P = 0.921		ρ = 0.088 / P = 0.350		ρ = 0.169 / P = 0.076	
No	100 (83)	23 (88)	73 (82)	14 (82)	85 (83)	12 (92)	82 (82)	14 (100)	78 (81)
Yes	20 (17)	3 (12)	16 (18)	3 (18)	17 (17)	1 (8)	18 (18)	0 (0)	18 (19)
Seminal vesicle invasion – no. (%)		ρ = -0.011 / P = 0.907		ρ = 0.085 / P = 0.351		ρ = -0.081 / P = 0.389		ρ = 0.074 / P = 0.437	
No	20 (17)	25 (96)	86 (96)	17 (100)	97 (95)	12 (92)	97 (97)	14 (100)	92 (96)
Yes	100 (83)	1 (4)	3 (4)	0 (0)	5 (5)	1 (8)	3 (3)	0 (0)	4 (4)
CAPRA-S risk group – no. (%)*		ρ = 0.085 / P = 0.492		ρ = 0.180 / P = 0.197		ρ = 0.032 / P = 0.817		ρ = 0.032 / P = 0.544	
Low	48 (40)	13 (50)	32 (36)	10 (59)	38 (37)	6 (46)	39 (39)	6 (43)	38 (40)
Intermediate	44 (37)	8 (31)	36 (40)	5 (29)	38 (37)	4 (31)	39 (39)	7 (50)	35 (36)
High	9 (8)	2 (7.5)	6 (7)	0 (0)	9 (9)	1 (7.5)	6 (6)	0 (0)	6 (6)
Missing	19 (15)	3 (11.5)	15 (17)	2 (12)	17 (17)	2 (15.5)	16 (16)	1 (7)	17 (18)
Biochemical recurrence – no. (%)		ρ = 0.142 / P = 0.128		ρ = 0.195 / P = 0.033		ρ = 0.028 / P = 0.763		ρ = 0.163 / P = 0.088	
Negative	78 (65)	20 (77)	54 (61)	15 (88)	63 (62)	9 (69)	65 (65)	12 (86)	60 (62.5)
Positve	42 (35)	6 (23)	35 (39)	2 (12)	39 (38)	4 (31)	35 (35)	2 (14)	36 (37.5)
Disease-free survival – no. (%)		ρ = -0.141 / P = 0.168		ρ = -0.202 / P = 0.045		ρ = -0.030 / P = 0.770		ρ = -0.170 / P = 0.106	
Yes	41 (34)	6 (23)	34 (38)	2 (12)	38 (37)	4 (31)	34 (34)	2 (14)	35 (36)
No	58 (48)	15 (58)	42 (47)	11 (65)	47 (46)	7 (53.5)	39 (39)	9 (64)	45 (47)
Missing	21 (18)	5 (19)	13 (15)	4 (23)	17 (17)	2 (15.5)	27 (27)	3 (22)	16 (17)

*The CAPRA-S scores were categorized to give the three risk groups: Low risk if sscore 0-2; Intermediate risk if score 3 to 5; High risk if score 6 to 12.

Spearsman´s correlation ρ (95% CI) / P value.

IQR, interquartile range; PSA, prostate-specific antigen; AR, androgen receptor.

**Table 3:** Correlation between PDHA complex components and androgen receptor protein expression in prostate cancer.

Patients - no.	N = 120	AR negative	AR positive
		(N = 26)	(N = 92)
Missing	2		
PDHA1- no. (%)		p = 0.195 / P = 0.035	
Negative	89 (75)	23 (88)	66 (72)
Positive	28 (24)	2 (8)	26 (28)
Missing	1 (1)	1 (4)	0 (0)
PDP1 - no. (%)		p = 0.184 / P = 0.046	
Negative	104 (88)	25 (96)	79 (86)
Positive	13 (11)	0 (0)	13 (14)
Missing	1 (1)	1 (4)	0 (0)
PDP2 - no. (%)		p = 0.124 / P = 0.180	
Negative	86 (73)	21 (80)	65 (70)
Positive	31 (26)	4 (15)	27 (30)
Missing	1 (1)	1 (4)	0 (0)
PDK1 - no. (%)		p = 0.282 / P = 0.003	
Negative	26 (22)	11 (42)	15 (16)
Positive	87 (74)	13 (50)	74 (81)
Missing	5 (4)	2 (8)	3 (3)
PDK2 - no. (%)		p = 0.299 / P = 0.001	
Negative	15 (13)	8 (30)	7 (7)
Positive	102 (86)	17 (65)	85 (93)
Missing	1 (1)	1 (4)	0 (0)
PDK3 - no. (%)		p = -0.046 / P = 0.625	
Negative	13 (11)	2 (8)	11 (12)
Positive	99 (84)	21 (80)	78 (85)
Missing	6 (5)	3 (12)	3 (3)
PDK4 - no. (%)		p = 0.206 / P = 0.031	
Negative	14 (12)	6 (23)	8 (9)
Positive	96 (81)	17 (65)	79 (86)
Missing	8 (7)	3 (12)	5 (5)

Spearsman's correlation p (95% Cl) / P value; AR, Androgen receptor.

**Figure 4:**
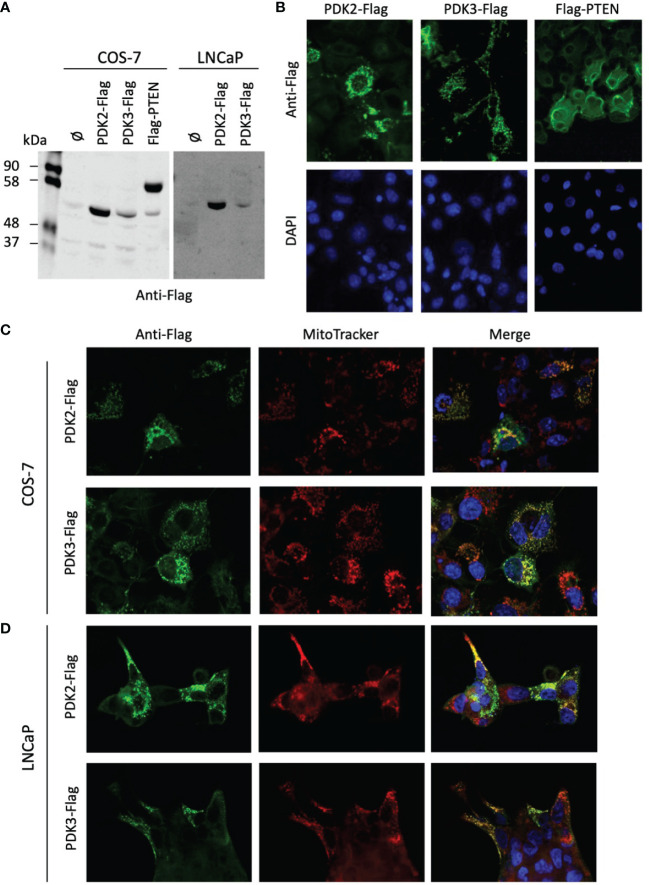
Expression and subcellular localization of PDK2 and PDK3 in PCa cells. **(A)** Immunoblot of ectopically expressed PDK2-Flag, PDK3-Flag, and Flag-PTEN (as a control) in COS-7 and LNCaP cells using anti-Flag antibody. **(B)** Immunofluorescence of PDK2-Flag, PDK3-Flag, and Flag-PTEN in COS-7 cells, using anti-Flag antibody (green). **(C)** Immunofluorescence of PDK2-Flag and PDK3-Flag (green) as in B, with Mitotracker as a mitochondria marker (red). **(D)** Immunofluorescence of PDK2-Flag and PDK3-Flag (green) in LNCaP cells, with Mitotracker as a mitochondria marker (red). In **(B–D)** nuclei were stained with DAPI (blue). Note the punctuated and mitochondrial localization of PDK2 and PDK3, as compared to the cytoplasmic PTEN localization.

PDH complex components are found at the mitochondria, but they have also been found in the nucleus, which has been proposed to have clinical implications (36, 43). Next, we investigated by immunoblot and immunofluorescence the expression and subcellular localization of PDK2 and PDK3 (tagged with a Flag epitope at the C-terminus) ectopically expressed in COS-7 (as a suitable cell model for ectopic protein expression) and LNCaP PCa cells (**Figures 4A–D**). The expression of the phosphatase PTEN was monitored as a control. PDK2-Flag and PDK3-Flag proteins displayed a predominant punctate pattern of expression that overlapped with Mitotracker marker staining, indicating a major mitochondrial localization in cells (**Figures 4B–D**). This is in accordance with the mitochondrial subcellular localization reported for PDH components in other human cancer cell lines (43). In contrast, Flag-PTEN displayed cytoplasmic/nuclear localization (**Figure 4B**). Together, these results illustrate differential subcellular localization of PDKs in cells and in PCa tissues. In the case of PDK2, which showed predominant nuclear localization in PCa tissues, further studies are required to elucidate how its localization at specific subcellular compartments in PCa tumors may affect PCa progression.

## Discussion

PCa cells show a unique metabolic reprogramming process during their progression towards malignancy, in which signaling through AR plays an essential role. Primary PCa tumor cells display unusual high oxidative respiration levels, which switch in CRPC cells to high aerobic glycolysis upon androgen-independent AR signaling (44). PDH enzymatic activity is a major universal driver of the energy metabolism in cells, coordinating the energy flux through the glycolytic and the mitochondrial TCA-oxidative pathways. Accordingly, PDH complex plays an important role in cancer-associated metabolic reprograming (27). Here, we have analyzed by IHC the expression of PDH components in PCa tumor samples. We have found a positive correlation of AR expression with PDHA1, PDP1, PDK1, PDK2, and PDK4 expression, which sustains the involvement of AR signaling in the control of PDH activity in PCa cells. In this regard, PDHA1 and PDK2 have been reported in a meta-analysis study as common androgen-regulated genes (45). In concordance, PDH/PDHA1 protein and its activator phosphatase PDP1 have been found to be overexpressed in PCa, in association with high Gleason score (36, 46), although low PDHA1 protein expression in PCa tumors has also been associated with poor prognosis (35). In our study, we found significant correlation of PDP1 expression, but not PDHA1, with stage and extracapsular extension. Prostate conditional *Pten*-null mice, knocked-out for PDHA1 expression in the prostate, displayed growth inhibition of prostate cells, and pharmacological inhibition of PDH activity in prostate *Pten*-null mice and in human PCa cells caused tumor and cell growth inhibition (36). Similarly, diminished cell growth was observed in PDHA1 knock-out LNCaP PCa cells (35, 47). Overall, these findings suggest a potential therapeutic benefit of PDH inhibition in advanced PCa tumors.

PDKs are physiologic negative regulators of PDH. In a variety of cancer types, PDK1-3 have been proposed to play oncogenic roles, whereas PDK4 has been proposed to play both oncogenic and tumor suppressive functions depending on the tumor type (33). In PCa, PDK1 has been found to be upregulated in correlation with disease progression, and PDK1 knock-down using siRNAs increased PCa cell migration and invasion, without significantly affecting cell proliferation (48). On the other hand, low PDK4 expression has been associated with biochemical recurrence in PCa datasets (49). PDK4 mRNA, followed by PDK2, are the more abundant PDK mRNAs detected in prostate and PCa (**Supplementary Figure 1**), and our IHC analysis revealed expression of all PDK proteins in PCa tumors. Notably, we detected correlation of PDK2 high expression with higher biochemical recurrence and lower disease-free survival, suggesting a pro-oncogenic role for PDK2 in PCa. This is in line with the proposed oncogenicity of PDK2 overexpression in other cancer types (50, 51). The tumor suppressor p53 negatively regulates PDK2 transcription (52), making of interest the analysis of the participation of p53 in the regulation of PDK2 expression in PCa cells. PDK2 showed a marked nuclear localization in PCa tumors, but not in PCa cell lines, which displayed PDK2 mitochondrial localization. Additional experiments are necessary to uncover the functional activities of nuclear PDK2 in PCa tissue.

The inhibition of PDKs by DCA, alone or in combination with other drugs, has been proposed as an alternative therapeutic anti-cancer approach, especially in chemoresistant tumors (34, 53–56). In our study, treatment of LNCaP and DU-145 PCa cells with DCA resulted in diminished cell proliferation, suggesting the feasibility of DCA, or DCA-related drugs, in the treatment of PCa. However, the clinical use of DCA in cancer therapy is limited, mainly due to undesired side effects, including peripheral neurotoxicity (32, 57). Kailavasan et al. reported metabolite ratios alterations in highly metastatic LNCaP-LN3 cells upon DCA treatment, which were not detected in poorly metastatic LNCaP cells (28). It has also been reported the sensitization to radiation of PCa cells by DCA (58), as well as PDK isozyme-specific effects of DCA on PCa cells (34). Interestingly, early studies on PDKs enzymatic activity revealed PDK2 as the PDK more efficiently inhibited by DCA (59). Whether DCA selectively targets PDK2 in PCa cells needs to be tested. It cannot be ruled out a DCA antiproliferative effect in PCa cells mediated by other PDKs. Dedicated studies are required to ascertain the involvement of inhibition of specific PDKs in the sensitivity to current anti-PCa therapies.

## Data Availability Statement

The original contributions presented in the study are included in the article/**Supplementary Material**. Further inquiries can be directed to the corresponding author.

## Ethics Statement

The studies involving human participants were reviewed and approved by Comité Ético de Investigación Clínica, Hospital Universitario Cruces, Barakaldo, Spain. The patients/participants provided their written informed consent to participate in this study.

## Author Contributions

CN-X, JL, and RP contributed to conception and design of the study. CN-X, JM, ME, KF-K, JL, and RP performed experiments, collected and analyzed data. CN-X and KF-K performed the statistical analysis. RL and JL provided tumor samples, data acquisition and clinical details of patients. CN-X and RP wrote the first draft of the manuscript. CN-X, JM, KF-K, GM, ØF, RL, JL, and RP contributed to manuscript revision, read, and approved the submitted version.

## Funding

This study was funded by Instituto de Salud Carlos III (ISCIII; Spain and The European Social Fund+ ( "Investing in your future"; Grant number CP20/00008), and The Research Council of Norway (Grant number 239813) and Marie Skłodowska‐Curie Actions, UNIFOR‐FRIMED Legacy (2020, Norway) to CN-X; and by Ministerio de Economía y Competitividad (Spain and The European Regional Development Fund; Grant number SAF2016‐79847‐R) to RP and JL. CN-X is the recipient of a Miguel Servet Research Contract from ISCIII; Grant number CP20/00008).

## Conflict of Interest

The authors declare that the research was conducted in the absence of any commercial or financial relationships that could be construed as a potential conflict of interest.

## Publisher’s Note

All claims expressed in this article are solely those of the authors and do not necessarily represent those of their affiliated organizations, or those of the publisher, the editors and the reviewers. Any product that may be evaluated in this article, or claim that may be made by its manufacturer, is not guaranteed or endorsed by the publisher.
