# Supplementary material for: Heterogeneous Expression and Subcellular Localization of Pyruvate Dehydrogenase Complex in Prostate Cancer
Source: Front Oncol. 2022 May 25;12:873516. doi: 10.3389/fonc.2022.873516 (PMC9174590; doi:10.3389/fonc.2022.873516)

Supplementary Figure legend

Supplementary Figure 1. mRNA expression of PDH genes in prostate tissues and prostate cancer. RNA-seq data from normal tissues are from the database GTEx (Genotype-Tissue Expression), and data are expressed as RPKM (reads per kilobase per million mapped reads). RNA-seq data from tumors are from database TCGA (The Cancer Genome Atlas), and data are expressed as FPKM (fragments per kilobase of exon per million reads). All data are from https://www.proteinatlas.org/.

Supplementary Figure 1

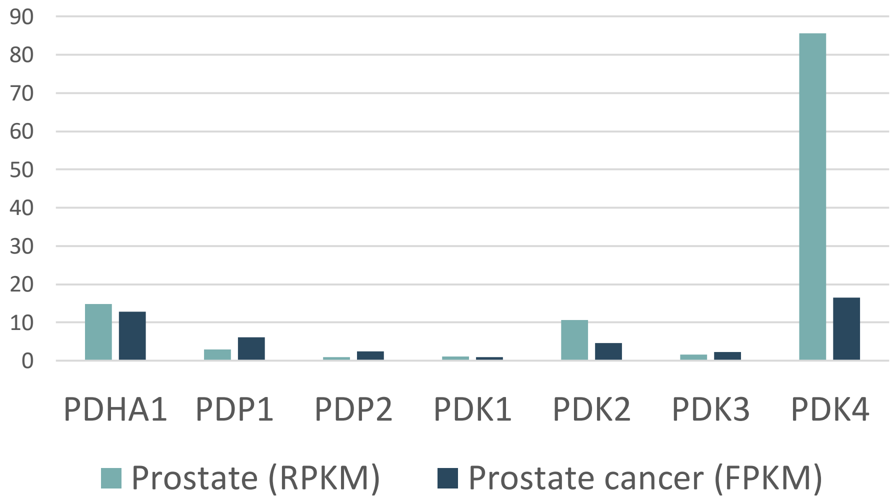

Supplement: Supplementary file 1 [file DataSheet_1.pdf]
